# Bypass Treatments for Primary Coenzyme Q10 Deficiency: An Update

**DOI:** 10.3390/ijms27083526

**Published:** 2026-04-15

**Authors:** David Mantle, Neve Cufflin, Iain P. Hargreaves

**Affiliations:** 1 Pharma Nord (UK) Ltd., Morpeth NE61 2DB, Northumberland, UK; dmantle@pharmanord.co.uk; 2 School of Pharmacy, Liverpool John Moores University, Liverpool L3 5UA, Merseyside, UK; n.m.cufflin@2025.ljmu.ac.uk

**Keywords:** coenzyme Q10, primary deficiency, bypass mechanisms, 4-hydroxybenzoic acid, 2,4-dihydroxybenzoic acid, vanillic acid, blood–brain barrier

## Abstract

Primary coenzyme Q10 (CoQ10) deficiency results from mutations in genes involved in the CoQ10 biosynthetic pathway. In humans, at least 10 genes (*PDSS1*, *PDSS2* to *COQ10*) are required for the biosynthesis of functional CoQ10, a mutation in any one of which can result in a deficit in CoQ10 status and present as primary CoQ10 deficiency. Furthermore, the genes *NDUFA9* and *HPDL*, whilst not part of the *PDSS1*, *PDSS2* to *COQ10* gene sequence, have also been shown to have a crucial role in CoQ10 biosynthesis. A major problem in treating primary CoQ10 deficiencies is the poor bioavailability of supplemental CoQ10, both in terms of lack of absorption from the digestive tract and inability to cross the human blood–brain barrier. Bypass strategies aim to circumvent this problem by using more bioavailable precursor analogues that can enter the cell and be incorporated into the CoQ10 synthesis pathway downstream of the affected enzyme, examples being 4-hydroxybenzoic acid, 2,4-dihydroxybenzoic acid or vanillic acid, which, in contrast to CoQ10, are small, water-soluble molecules. In this article, we have, therefore, reviewed potential bypass mechanisms for primary CoQ10 deficiencies, PDSS1, PDSS2 to COQ10, together with NDUFA9 and HPDL, using such precursors. Most of the published data relating to the bypass therapy of primary CoQ10 deficiency is derived from cell lines or animal models, and few human studies have so far been undertaken. In addition, further research is required to investigate the potential mechanisms by which bypass compounds such as 4-HB may access the human blood–brain barrier (BBB), for example, using in vitro co-culture BBB model systems incorporating CoQ10-deficient neurons. Overall, the objective of this article is, therefore, to systematically review the available data for each of the primary CoQ10 deficiencies, PDSS1, PDSS2 to COQ10 together with NDUFA9 and HPDL, in particular to identify the clinical potential of such studies.

## 1. Introduction

**Figure 1:**
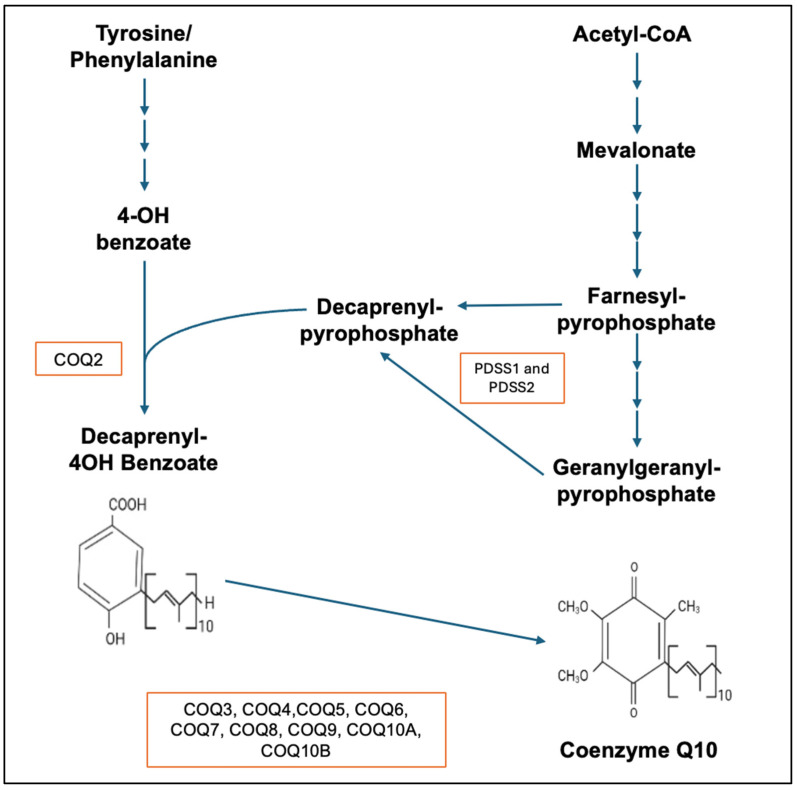
The coenzyme Q10 biosynthetic pathway showing the enzymes PDSS1 and PDSS2–COQ10B, which are involved in the synthesis of CoQ10.

Coenzyme Q10 (CoQ10) has a number of key functions in supporting normal cell metabolism; principally, its role is in cellular energy supply/ATP synthesis via mitochondrial oxidative phosphorylation, but it also acts as a major endogenously synthesised lipid-soluble antioxidant, protecting cellular/sub-cellular organelle membranes from free-radical-induced oxidative damage. CoQ10 also has important roles in the metabolism of lysosomes, sulphides, amino acids, pyrimidine, and cholesterol, as well as a role in directly mediating the expression of more than one hundred genes, including those involved in the inflammatory process. Structurally, CoQ10 comprises a benzoquinone ring, which confers the redox properties of the molecule, and a polyisoprenoid tail, which is responsible for its lipophilicity [1]. Given its key role in normal cellular metabolism, it is unsurprising that deficiency of CoQ10 has been implicated in the pathogenesis of a number of disorders. Deficiency of CoQ10 is broadly divided into two types, primary and secondary, and this article is focussed on primary CoQ10 deficiency. Primary CoQ10 deficiency results from mutations in genes involved in the CoQ10 biosynthetic pathway. In humans, at least 10 genes (*PDSS1*, *PDSS2* to *COQ10*) are required for the biosynthesis of functional CoQ10, a mutation in any one of which can result in a deficit in CoQ10 status. The biosynthesis of CoQ10 is a complex, multistep process that takes place in various sub-cellular locations (Figure 1). The polyisoprenoid tail is synthesised (via polyprenyl diphosphate synthase) in the cytosol via the mevalonate pathway, with attachment to the benzoquinone ring (originating from tyrosine and its metabolite 4-hydroxybenzoate) taking place within the mitochondria. The ring structure is then further modified via hydroxylation, methylation, and decarboxylation by a set of enzymes grouped in a complex (Complex Q in humans; Q syndrome in yeast) [2]. Complex Q comprises proteins COQ3–COQ9, together with various cofactors, and is associated with the matrix face of the inner mitochondrial membrane; it organises the individual synthetic enzymes in a way that maximises synthetic efficiency while preventing the escape of non-functional intermediate metabolites such as demethoxy-ubiquinone (DMQ) [3]. For a bypass strategy to be successful, Complex Q must maintain some structural stability; the effectiveness of these bypass strategies is generally limited to COQ mutations that disrupt enzymatic activity but still maintain the structural stability of Complex Q. Much of the data relating to the biosynthesis of CoQ10 were originally obtained from studies of yeast, with CoQ10 biosynthesis subsequently shown to be highly conserved in yeast (involving genes *Coq1* to *Coq11*) and humans (involving genes *PDSS1*, *PDSS2* to *COQ10*) [2].

Symptomatic improvement following supplementation with CoQ10 has been reported in some types of primary CoQ10 deficiency, notably those involving peripheral tissues such as muscle or kidneys, but has been less successful in neurological disorders; this, in turn, may be linked to difficulty CoQ10 has in crossing the human blood–brain barrier, as well as the more general low bioavailability of supplemental CoQ10 [4]. This is a reflection of a more general problem relating to the potential role of CoQ10 in the treatment of neurological disorders; there is evidence for mitochondrial dysfunction, oxidative stress and inflammation in a number of neurological disorders and, therefore, a rationale for CoQ10 therapy based on the cellular functions outlined above. However, because of the difficulty of access of CoQ10 across the human BBB, the results of clinical trials supplementing CoQ10 in neurological disorders have, in general, been disappointing. There is, therefore, an urgent need to improve the access of CoQ10 across the human BBB, not just for the treatment of neurological manifestations of primary CoQ10 deficiency, but for neurological disorders in general. One of the potential methods of addressing this problem is CoQ10 bypass therapy, and this approach is the subject of the present article.

An alternative approach to treating primary CoQ10 disorders involves supplementation with CoQ10 precursors, providing chemical groups lacking due to defects in specific enzymes. Most of the published data relating to the bypass therapy of primary CoQ10 deficiency is derived from cell lines or animal models. The objective of this article was, therefore, to systematically review the available data for each of the primary CoQ10 deficiencies, COQ1 to COQ10, in particular to identify the clinical potential of such studies; this article, therefore, serves as a detailed update to the commentary article by Herebian et al. published in 2018 [5]. Studies were identified from the Medline database of the National Library of Medicine, using the search keywords listed following the abstract of this article.

## 2. Bypass Mechanisms for Primary CoQ10 Deficiencies: PDSS1, PDSS2–COQ10

**Figure 2:**
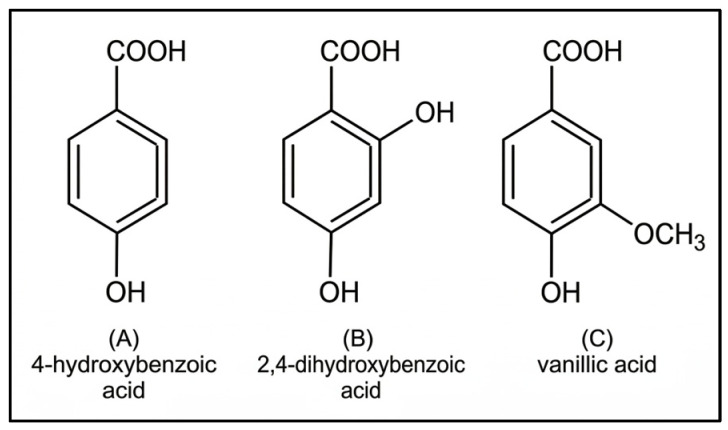
Chemical structures of synthetic ring precursors used to bypass coenzyme Q deficiencies. (**A**) 4-hydroxybenzoic acid, (**B**) 2,4-dihydroxybenzoic acid and (**C**) vanillic acid.

The background to individual COQ10 deficiencies described in the following section is described in more detail in the review by Mantle et al. [2], from which the following summaries of disease characteristics are derived. Supplementation with structural analogues of hydroxybenzoic acid (a precursor of the benzoquinone ring) has been proposed as a bypass mechanism for several types of primary CoQ10 deficiency since these derivatives already possess hydroxyl or methoxy groups in variable positions on the aromatic ring of relevance to various steps in CoQ10 biosynthesis, i.e., 2-hydroxybenzoic acid (or salicylic acid, 2-HB), 4-hydroxybenzoic acid (4-HB), 2,4-dihydroxybenzoic acid (2,4-DHB or β-resorcylic acid), 3,4-dihydroxybenzoic acid (or protocatechuic acid, 3,4-DHB), and 4-hydroxy-3-methoxybenzoic acid (or vanillic acid; Figure 2).

The *PDSS1* and *PDSS2* genes encode subunits 1 and 2, respectively, of the enzyme hexaprenyl pyrophosphate synthetase, which catalyses the first step in CoQ10 biosynthesis, the synthesis of the polyisoprenoid tail. Mutations in *PDSS1* reportedly result in steroid-resistant nephrotic syndrome, encephalopathy, and optic nerve atrophy. To date, a total of six patients with *PDSS1* mutations have been identified [2]. CoQ10 deficiency resulting from mutations in the *PDSS2* gene is particularly associated with neonatal/infantile-onset renal disease, with variable neurological involvement. To date, a total of seven patients with *PDSS2* mutations have been described [2]. No studies investigating potential bypass mechanisms in PDSS1 or PDSS2 deficiency have been identified. The lack of potential bypass treatments for PDSS1/PDSS2 deficiency is a consequence of PDSS1/PDSS2 in the synthesis of the polyisoprenoid tail, and its position is the first step of CoQ10 biosynthesis; because of these factors, it is mechanistically very difficult to design appropriate bypass treatments.

The *COQ2* gene encodes the enzyme p-hydroxybenzoate–polyprenyl transferase, which catalyses the conjugation of the benzoquinone ring with the decaprenyl side chain in CoQ10 biosynthesis. *COQ2* mutations typically manifest as encephalopathy and nephropathy of variable severity. A total of 63 patients with a COQ2 deficiency have been identified to date [2]. Bypass mechanisms for COQ2 deficiency involve supplementation with 4-HB, as a precursor of the benzoquinone ring structure of CoQ10. High concentrations of exogenous 4-HB have been shown to fully restore CoQ10 levels and mitochondrial function and rescue severe multisystemic disease and lethality in relevant mouse models and patient cell cultures. Thus, Corral-Sarasa et al. [6] found that supplementation with 4-HB reduced multisystemic disease and prevented perinatal lethality in *Coq2* mutant mice. These mice had the homozygous *Coq2A252*V pathogenic variant (c.755C>T) and manifested severe multisystemic disease, including cardiac insufficiency, oedema, neurodevelopmental delay, and perinatal lethality. This mutation is homologous to the human *p.Ala302Val* (*A302V*) pathogenic variant found in patients with severe infantile-onset COQ2 deficiency. The treatment with 4-HB successfully increased residual CoQ10 levels, normalised mitochondrial function, and improved outcomes for severely affected tissues, including the heart and brain, a result that CoQ10 supplementation failed to fully achieve. Similarly, supplementation with 4-HB fully restored endogenous CoQ10 biosynthesis in partially deficient COQ2 human fibroblasts harbouring homozygous mutant alleles, including the A302V severe allele. The *A302V* mutation is responsible for infantile multisystem disease, characterised by neonatal onset, rapid clinical decline, seizures, and multi-organ failure. The *A302V* mutation also results in a substantial, but not total, reduction in CoQ10 biosynthesis, resulting in CoQ10 levels of 35–45% of normal. Such partially deficient cells exhibit high levels of reactive oxygen free radical species production, severe mitochondrial dysfunction, and accelerated cell death, particularly in the presence of stress. Following treatment with 4-HB, mitochondrial bioenergetics are improved, and cell viability under oxidative stress is restored [7]. Supplying high levels of 4-HB may force a still partially functional mutated COQ2 protein to work more efficiently by overcoming transport and binding issues caused by the mutation. Pathogenic point mutations in the *COQ2* gene, while causing only small global changes to the overall protein structure, can induce alterations in the hydrophobic pocket surrounding the active site, hindering access of the 4-HB and decaprenyl chain substrates [7].

Based on data from preclinical studies above, Distelmaier et al. [8] described treatment with 4-HB in a 3-year-old with COQ2 deficiency that had not responded to supplementation with high-dose CoQ10. A first-in-humans individual therapeutic trial was initiated, supplementing 4-HB in the 3-year-old with genetically confirmed primary CoQ10 deficiency due to compound heterozygous pathogenic *COQ2* variants. The authors of this study reported that “the patient presented with a Leigh-like syndrome characterised by bilateral brain lesions, developmental delay, muscular hypotonia, failure to thrive, lactic acidosis, and steroid-resistant nephrotic syndrome. Despite high-dose oral CoQ10 supplementation, the clinical response had been minimal. Following the initiation of oral 4-HB treatment, the patient experienced rapid and sustained remission of proteinuria, improved renal hyperfiltration, and a gradual increase in serum CoQ10 concentrations. No adverse effects were observed during a six-month follow-up. Clinically, the patient showed notable improvements in motor skills, language acquisition, cognitive alertness, and overall development, accompanied by significant gains in growth and nutritional status. Clinical recovery was also reflected by improved scores on the Newcastle Paediatric Mitochondrial Disease Scale.”

The *COQ3* gene encodes a methyltransferase enzyme that catalyses both of the O-methylation steps in CoQ10 biosynthesis. To date, there have been no clinical studies reported in the medical literature relating to *COQ3* mutations. Although disease-causing mutations in the *COQ3* gene have not been reported, supplementation with 2,3-dimethoxy-4-hydroxybenzoic acid might, in principle, bypass the requirement for the respective active enzyme. Thus, the COQ3 enzyme is essential for converting 3,4-dihydroxy-5-polyprenylbenzoic acid into 3-methoxy-4-hydroxy-5-polyprenylbenzoic acid. By providing 2,3-dimethoxy-4-hydroxybenzoic acid, the metabolic requirement for the COQ3 enzyme is theoretically bypassed; however, at present, this remains a hypothetical issue.

The *COQ4* gene encodes a multienzyme complex organisation/stabilisation enzyme localised to the matrix side of the mitochondrial inner membrane, which, when deficient, results in CoQ10 deficiency, causing childhood-onset neurodegeneration. Recently, COQ4 has been reported to catalyse the oxidative decarboxylation of the C1 carbon of CoQ10 precursors [9]. A total of 44 patients with COQ4 deficiency have been identified to date [2]. In a COQ4-deficient cell line, supplementation with 2,4-DHB or vanillic acid failed to alleviate COQ4 deficiency [7]. This may be mainly because of the structural function of COQ4 rather than its decarboxylase activity, which is required to stabilise the CoQ multienzyme complex (Q-synthome). Because COQ4 provides structural integrity to the complex, its absence prevents the proper assembly of the biosynthetic machinery. Consequently, providing unnatural head group precursors, such as 2,4-DHB or vanillic acid, cannot bypass the structural failure of the complex, making them ineffective treatments for *COQ4* defects.

The *COQ5* gene encodes the methyltransferase enzyme responsible for the C-methyltransferase step in CoQ10 biosynthesis (i.e., conversion of 2 methoxy-6-polyprenyl-1,4-benzoquinol to 2 methoxy-5-methyl-6-polyprenyl-1,4 benzoquinol). To date, there has been only one clinical study reported relating to the mutation of the *COQ5* gene in three siblings, who partially responded to supplementation with CoQ10 [10]. The patients, who presented with early-onset cerebellar ataxia, encephalopathy, seizures, and cognitive disability, were found to have a homozygous 9590 bp duplication in the *COQ5* gene, which caused a reduction in CoQ10 levels in white blood cells and muscle tissue. Supplementation with CoQ10 (15 mg/kg/day for 6 months) resulted in a significant improvement in their ICARS (International Cooperative Ataxia Rating Scale) scores. In principle, supplementation with 2-methyl-4-hydroxybenzoic acid (2,4-DHB) may provide a bypass mechanism for this enzymic defect. This is because COQ5 is an S-adenosyl methionine (SAM)-dependent C-methyltransferase that adds a methyl group to the 5-position of the benzoid ring in the CoQ10 biosynthesis pathway. 2-methyl-4-hydroxybenzoic acid already contains the methyl group at the position that would normally be added by the COQ5 enzyme. When supplied to cells, this compound can be prenylated by COQ2 and processed by downstream enzymes into functional CoQ10, bypassing the requirement for COQ5-mediated methylation. As with the situation described for COQ3 above, at present, this remains a hypothetical issue.

The *COQ6* gene encodes an enzyme (a flavin-dependent monooxygenase) that is responsible for the C5-hydroxylation of the quinone ring during CoQ10 synthesis. Mutations in this gene result in primary CoQ10 deficiency-6, an autosomal recessive disorder that typically manifests as a progressive infantile-onset steroid-resistant nephrotic syndrome resulting in end-stage renal failure, together with sensorineural deafness; individuals may also be susceptible to the development of Schwannomatosis, a form of neurofibromatosis characterised by the formation of benign tumours in the nervous system. A total of 45 patients with COQ6 deficiency have been identified to date [2]. Doimo et al. [11] demonstrated that supplementation with vanillic acid or 3,4-DHB (two analogues of 4HB that carry either a methoxyl or a hydroxyl group in position 5 of the ring) can bypass the need for the COQ6 enzyme, restoring CoQ10 biosynthesis and respiratory growth in Δcoq6 yeast cells (strains of Saccharomyces cerevisiae in which the *COQ6* gene has been deleted, as indicated by the Δ symbol). Δcoq6 cells suffer from severe mitochondrial dysfunction and reduced ATP production and are typically unable to grow on non-fermentable carbon sources because they cannot perform oxidative phosphorylation. Similarly, Ozeir et al. [12] demonstrated that supplementary vanillic acid or 3,4-DHB bypass the COQ6 defect in mutant yeast, restoring CoQ10 biosynthesis and respiratory growth. Widmeier et al. [13] found that administration of 2,4-DHB in podocyte-specific COQ6-deficient mice prevented renal dysfunction, including proteinuria and focal segmental glomerulosclerosis, by bypassing the COQ6 enzyme in CoQ10 biosynthesis, with survival rates significantly improved. Using a COQ6-deficient human cell line (HEK 293) generated via CRISPR/Cas9 technology, supplementation with vanillic acid recovered CoQ10 synthesis and ATP production and restored oxidative stress to normal levels [14]. Based on data from the above studies, patients with COQ6 deficiency may, therefore, respond to supplementation with vanillic acid or 3,4-dDHB, although this remains to be investigated.

The *COQ7* gene encodes the enzyme 5-demethoxyubiquinone hydroxylase, which catalyses the hydroxylation and conversion of demethoxyubiquinone to 5-hydroxy-ubiquinone. Mutations in the *COQ7* gene result in primary COQ7. To date, four clinical cases relating to mutations in the *COQ7* gene have been reported [2]. In individuals with pathogenic *COQ7* mutations, CoQ10 levels are severely reduced, and demethoxyubiquinone (DMQ) accumulates. The primary bypass mechanism for deficiencies in the COQ7 enzyme is the use of the structural analogue 2,4-dihydroxybenzoic acid (2,4-DHB, also known as resorcyclic acid), as 2,4-DHB already has a hydroxyl group at the C6 position of the benzoquinone ring, the position where COQ7 typically acts. This compound, therefore, provides an alternative precursor for CoQ10 synthesis, effectively circumventing the need for the specific hydroxylation step normally catalysed by COQ7. Experiments using patient-derived fibroblasts with COQ7 deficiency (p.Val141Glu variant) have shown a significant improvement of CoQ10 levels and mitochondrial function following 2,4-DHB treatment [15]. The efficacy of 2,4-DHB treatment is dependent on the severity of the *COQ7* mutation; while highly effective in p.Val141Glu cells, it has been shown to be less effective or not effective in less severe *COQ7* variants, such as p.Leu111Pro, as noted in the following study. Thus, Wang et al. [16] investigated two patients with different *COQ7* mutations (V141E and L111P) differing in the severity of symptoms (V141E, more severe; L111P, less severe). Treatment of fibroblast cell lines from the two patients with 2,4 DHB showed restoration of CoQ10 synthesis and improved mitochondrial function in the more severe V141E mutation, while cells carrying the less severe L111P mutation showed no improvement in these parameters, potentially because the native pathway still had some activity, leading to competitive effects. Thus, in cells with milder mutations, where there is still some significant residual COQ7 activity, 2,4-DHB competes with the natural pathway precursor (4-HB) in the synthetic pathway; by supplying 2,4-DHB, the small amount of natural CoQ10 production is inhibited, often resulting in no net gain or even a decrease in total CoQ10 levels. Gonzalez-Garcia et al. [17] reported improved sulphide metabolism following co-administration of CoQ10 and vanillic acid in fibroblasts from two patients with COQ7 deficiency resulting from different mutations in the *COQ7* gene. CoQ10 has a key role in sulphide metabolism as a cofactor of the enzyme sulphide quinone oxidoreductase, which reduces levels of toxic hydrogen sulphide, which can contribute to oxidative stress [18]. In yeast with Coq7 deficiency, 2,4-DHB supplementation successfully reduced oxidative-related growth defects [19]. In conditional *Coq7* knockout mice, administration of 2,4-DHB reversed several severe disease phenotypes, including growth retardation, weight loss, fur loss, and a shortened lifespan [20]. Conditional *Coq7* knockout mice are genetically engineered to switch off the *Coq7* gene in a specific tissue from birth; this approach is essential because a full-body (constitutive) deletion of *Coq7* is embryonically lethal.

The *COQ8A* gene, also known as *ADCK3* or *CABC1*, encodes an atypical protein kinase that stabilises the multi-protein CoQ synthesis complex. Q-syndrome patients with mutations in this gene typically develop childhood-onset cerebellar atrophy and ataxia and have decreased CoQ10 content in their muscle, fibroblast, and lymphoblast cells. A total of 123 patients with COQ8A deficiency have been identified [2]. The *COQ8B* gene (also known as *ADCK4*) similarly encodes an atypical protein kinase, a variant which results in steroid-resistant nephrotic syndrome with variable neurological involvement (presenting mainly in childhood or adolescence). To date, 99 patients with *COQ8B* mutations have been identified [2]. Treatment of 3-month-old *ADCK4*-mutated mice with 2,4-DHB prevented the development of renal pathology and reversed mitochondrial dysfunction [21]. When administered via drinking water before severe symptoms developed, 2,4-DHB treatment prevented renal disease progression, improved kidney function and plasma albumin levels, and resulted in a normal survival rate comparable to control mice. At the cellular level, 2,4-DHB treatment was able to partially restore reduced mitochondrial respiratory chain complex II-III activity and rescue mitochondrial dysfunction in *ADCK4*-knockout podocytes.

The *COQ9* gene encodes a lipid-binding protein that is responsible, via its interaction with other COQ-encoded enzymes (particularly COQ7), for stabilising the COQ10 biosynthetic complex. Mutations in this gene have been reported in seven patients to date [2]. Luna-Sanchez et al. [22] found that 2,4-DHB treatment efficacy in *Coq9* mutant mice depends on the stability of the COQ multiprotein complex, specifically rescuing CoQ10 deficiency in *Coq9R239X* mice but not in *Coq9Q95X* mice. *Coq9R239X* mice are characterised by severe widespread CoQ10 deficiency associated with fatal encephalomyopathy and have responded to 2,4-DHB, increasing CoQ10 levels. In contrast, *Coq9Q95X* mice exhibit mild CoQ10 deficiency, manifesting as a reduction in mitochondrial respiratory chain complex I/III activity and mitochondrial respiration in skeletal muscle, as well as late-onset mild mitochondrial myopathy, which does not respond to 2,4-DHB. *Coq9R239X* mice respond to 2,4-DHB treatment because their specific mutation causes a severe, stable defect in CoQ10 biosynthesis that 2,4-DHB can bypass. This treatment works by both increasing CoQ10 levels and, more importantly, reducing the accumulation of DMQ9, a precursor that becomes toxic to mitochondrial function when it accumulates, as seen in *Coq9R239X* mice. Conversely, *Coq9Q95X* mice do not show the same response because they have lower levels of demethoxyubiquinone (DMQ) accumulation and a less severe, different pathogenic phenotype, suggesting that the 2,4-DHB mechanism is not effective for their specific metabolic state. The DMQ9/CoQ9 ratio (the toxic-to-functional ratio) in *Coq9Q95X* mice is significantly lower than in *Coq9R239X* mice; because their DMQ levels are not as severely elevated, the therapeutic benefit of reducing DMQ via the 2,4-DHB bypass is significantly less pronounced or effective, resulting in no positive response to the treatment. Gonzalez-Garcia et al. [17] reported that co-administration of CoQ10 and vanillic acid synergistically improved the survival and motor function of *Coq9R239X* mice via complementary mechanisms; CoQ10 enhanced peripheral quinone pools and modulated hepatic one-carbon metabolism, while vanillic acid reduced DMQ accumulation and neuroinflammation. In addition, vanillic acid supplementation improved cell viability and stimulated CoQ10 biosynthesis in human fibroblasts with *COQ9* mutations [7].

The success of these bypass therapies can be genotype-dependent, as the treatment’s efficacy is influenced by the degree of destabilisation of the overall CoQ10 multiprotein complex caused by the specific *COQ9* mutation.

The *COQ10A* gene is located on Chromosome 12 and comprises six exons; *COQ10B* is located on Chromosome 2 and comprises six exons. The *COQ10A* and *COQ10B* genes encode lipid-binding proteins that act as molecular chaperones, directing CoQ10 molecules to their final placement within membranes and are not directly involved in biosynthesis. COQ10A and COQ10B proteins are expressed in all tissues, although COQ10A is predominantly expressed in the heart and skeletal muscle. Polymorphisms in *COQ10A* or *COQ10B* have been implicated in predisposing patients to statin-associated myopathy. To date, there have been no clinical studies published relating to *COQ10A* or *COQ10B* variants; similarly, no studies were identified relating to potential bypass strategies for COQ10A or COQ10B.

Whilst not part of the *PDSS1*, *PDSS2*–*COQ10* gene mutation series described above, the enzyme 4-hydroxyphenylpyruvate dioxygenase-like protein (HPDL) encoded by the *HPDL* gene has also been shown to have a role in CoQ10 biosynthesis [23]. Patients with HPDL deficiency have been reported by Husain et al. ([24]; 17 individuals), Wiessner et al. ([25]; 34 individuals), Wang et al. ([26]; one individual), and Micule et al. ([27]; two individuals). The patient ages in these studies ranged from 6 months to 39 years; the clinical presentation typically included developmental delay, seizures, and spasticity. Treatment of patients with HPDL mutations with 4-hydroxymandelate (4-HMA, a metabolite of HPDL and precursor of 4-HB) or CoQ10 may stabilise or ameliorate some of these symptoms. To investigate whether 4-HMA or 4-HB supplementation promotes CoQ10 headgroup synthesis in vivo, Shi et al. [28] administered 4-HMA and 4-HB to *Hpdl*−/− mice. These genetically engineered homozygous knockout mice lack HPDL and model an ultra-rare, lethal mitochondrial encephalopathy in humans. *Hpdl*−/− mice appear normal at birth but develop severe motor defects (spastic paresis), epilepsy-like seizures, and significant weight loss, resulting in death by postnatal day 15. Both 4-HMA and 4-HB were incorporated into CoQ9 and CoQ10 in the brains of *Hpdl*−/− mice. The treatment restored motor function and reversed fatal CoQ10 deficiency symptoms, enabling a normal lifespan of 18 months or more compared to 15 days for untreated subjects. Furthermore, in a first-in-humans single-patient study, 4-HB treatment stabilised and improved the neurological symptoms of an 8-year-old child with progressive spasticity due to biallelic *HPDL* variants. 4-HB was administered in doses ranging from 25 mg/kg to 100 mg/kg at various time points over the treatment course of 250 days; the treatment was, in general, well tolerated. The treatment regime resulted in stabilisation of the child’s rapidly progressing spasticity, followed by objective and subjective improvement in the child’s motor function.

In a similar manner, whilst not part of the *PDSS1*, *PDSS2*–*COQ10* gene sequence responsible for the primary synthesis of CoQ10, the *NDUFA9* gene encodes a subunit of complex I (NDUFA9), and mutations in this gene disrupt complex I formation, in turn affecting CoQ10 formation by destabilising the CoQ10 biosynthesis complex, resulting in severe neurodevelopmental issues [29]. Naphthoquinones, such as menadione (vitamin K3) and chimaphilin, bypass NDUFA9 deficiency by acting as alternative electron carriers in the mitochondrial respiratory chain, accepting electrons upstream of deficient mitochondrial respiratory chain complex I and donating them to complex III to enable ATP production [30].

## 3. Bypass Treatments and the Blood–Brain Barrier

Studies supplementing CoQ10 for the treatment of neurological disorders associated with primary CoQ10 deficiency have, in general, reported disappointing outcomes, and this, in turn, likely results from the difficulty of exogenous CoQ10 to access the human BBB [4]. It is assumed that, as small water-soluble molecules, precursors such as 4-HB should be able to access the BBB; although, administration of 4-HB has been shown to increase cerebral CoQ10 levels in mice [6], however, to date, there have been no studies to confirm the BBB’s access to 4-HB in humans. Because of species differences in structure and function, animal models of the BBB may not be appropriate for the study of the BBB’s metabolite access in humans, and the use of in vitro cell-based models may be more appropriate [31].

An ideal BBB in vitro model for use in assessing the ability of CoQ10 precursors to penetrate the human BBB would be the human primary brain endothelium, but robust and reliable human cultures with sufficient barrier “tightness” to measure trans-endothelial transport do not currently exist. The use of the human primary brain endothelium as an ideal BBB in vitro model is severely limited by the inability to consistently produce cultures with high trans-endothelial electrical resistance (i.e., “tightness”) and robust, reliable, and scalable characteristics. While primary human brain microvascular endothelial cells (BMECs) are preferred for their biological relevance, they often lose their specific phenotypic characteristics (such as tight junctions and high permeability resistance) during isolation and cultivation.

In the absence of a human BBB model, a porcine BBB model may be appropriate; the porcine genome, anatomy, physiology and disease progression in general are closer to those in humans than in smaller laboratory animals [32]. The porcine model expresses a similar array of proteins compared to the human BBB, including the tight junction proteins claudin-5, occludin and ZO-1, functional BBB transporters, receptors and enzymes and forms a tight barrier, as assessed by a high trans-endothelial electrical resistance of 600–1000 Ω cm^2^ and the very low paracellular permeability of vascular markers [32,33].

Porcine brain endothelial cells (PBECs) are isolated and cultured to confluence on collagen/fibronectin-coated transwell filters [32]. Filters with confluent PBECs are suspended in culture plates above a neuronal cell line to establish co-cultures. CoQ10 precursors can then be applied above the PBECs and media below the PBEC filter sampled for analysis. Samples are taken at intervals to calculate BBB permeability coefficients and determine the capacity to cross the endothelial barrier to the neuronal cells below [34]. Human SHS-S5Y neuroblastoma cells can be used as the neuronal cells in this model, and the effect of the CoQ10 precursors applied above the BBB on neuronal CoQ10 status can be assessed. Furthermore, by treating neuronal cells or PBECs with para-aminobenzoate, CoQ10 deficiency can be induced into either the BBB or the neuronal cells, or both, as reported in a study by Wainwright et al. [34].

## 4. Discussion

The most obvious bypass mechanism for CoQ10 deficiency is supplementation with CoQ10 itself, and this strategy has resulted in significant clinical improvement in a number of secondary CoQ10 deficiency disorders, particularly heart failure [35]. However, supplementation with CoQ10 in neurological disorders associated with secondary CoQ10 disorders have been less successful, and this may be linked to difficulty in supplemental CoQ10 accessing the human blood–brain barrier; this in turn has focussed attention on the possibility of promoting endogenous CoQ10 synthesis in brain tissue via supplementation with CoQ10 precursors such as 4-HB or its analogues, which are known to access the human blood–brain barrier.

With regard to primary CoQ10 deficiency disorders, some of these can be treated by supplementation with CoQ10, particularly when diagnosed sufficiently early; however, the response to therapy depends on which COQ gene has been affected, and the particular location of the mutation within each of the respective genes. Thus, symptomatic improvement following supplementation with CoQ10 has been reported in some types of primary CoQ10 deficiency, notably those involving peripheral tissues such as muscle or kidneys, but has been less successful in those affecting the CNS; this again has focussed attention on developing bypass mechanisms based on supplementation with CoQ10 precursors, as reviewed in the present article. Most studies on developing bypass mechanisms for primary CoQ10 deficiency, to date, have been carried out using animal models or cultured cells from CoQ10-deficient patients; however, such studies have identified beneficial outcomes based on supplementation with 4-HB or its analogues in COQ2, COQ6, COQ7, COQ9 and HPDL deficiencies. The use of such bypass strategies in clinical practice is still at a very early stage, although recent reports by Distelmaier et al. [8], Shi et al. [28] and Mero et al. [36] on the successful treatment of CoQ10-deficient individuals demonstrate the therapeutic potential of bypass strategies for the future. As noted earlier in this article, 4-HB is regarded as a starting point in the synthesis of the CoQ10 benzoid ring structure, but the mechanism by which 4-HB originates from tyrosine is not completely understood; it is known that tyrosine aminotransferase catalyses the transamination of tyrosine into 4-hydroxyphenylpyruvate (4-HPP), but the overall sequence of steps via which tyrosine is converted into 4-HB has still to be fully elucidated in humans. Thus, mutations in genes encoding enzymes (possibly up to six) involved in the conversion of tyrosine into 4-HB can be a potential source of primary CoQ10 deficiency, an example being deficiency of the enzyme HPDL, which converts 4-HPP to 4-hydroxymandelate (4-HMA) during 4-HB synthesis, as noted earlier in this article. Subsequent steps requiring conversion of 4-HMA to 4-hydroxybenzaldehyde (4-Hbz) and 4-Hbz to 4-HB have still to be fully characterised in humans [37]. In addition, precursors such as 4-HB are polar (negatively charged) and cannot passively cross the inner mitochondrial membrane; the particular transporters that move these precursors from the cytoplasm into the mitochondrial matrix are presently unknown.

It is of note that, in addition to its role as a precursor in CoQ10 synthesis, 4-HB itself has significant antioxidant and anti-inflammatory action independent of CoQ10 [38]. Thus, 4-HB can activate the transcription factor Nrf2, with subsequent upregulation of antioxidant enzymes such as catalase and superoxide dismutase; 4-HB can also inhibit the action of NfkB, thereby reducing the production of pro-inflammatory cytokines. These antioxidant and anti-inflammatory activities may contribute to the beneficial action of 4-HB (in addition to its role in bypassing defects in CoQ10 synthesis) in patients with primary CoQ10 deficiencies, although this has still to be confirmed. Similarly, 2,4-DHB, 3,4-DHB and vanillic acid also have antioxidant and anti-inflammatory action [39,40]. It is also of note that substances such as resveratrol, kaempferol and coumarate can be converted into 4-HB, as precursors of the benzoid ring during CoQ10 synthesis in human cells [41,42,43].

With regard to safety, no randomised controlled clinical trials were identified supplementing 4-HB, 2,4-DHB, 3,4-DHB or vanillic acid in primary CoQ10 deficiency, or any other type of indication. However, several other types of studies have investigated the safety of 4-HB in human subjects. For example, 4-HB has been administered orally to adult humans at high doses, with individuals receiving 5g every 6 h over a 24 h period, without significant adverse effects [44]. There have been no specific studies in which 4-HB has been administered directly to children. However, the metabolism of 4-HB in children has been investigated as the primary metabolite of parabens (4-hydroxybenzoate esters), which are commonly found in children’s products; the level of 4-HB in children’s urine indicates widespread exposure [45]. In addition, following studies in rats, 4-HB, 2,4-DHB, 3,4-DHB and vanillic acid are considered to be of low toxicity; in rats, oral LD50 values > 2000 mg/kg have been reported for 4-HB, 3,4-HB, and vanillic acid and >3000 mg/kg for 2,4-DHB [44,46]. 4-HB, 2,4-DHB, 3,4-DHB and vanillic acid are all used as food additives, for example, as flavouring, antioxidant and antimicrobial agents. It is of note that 4-HB is also found in various types of food, with an estimated intake of 100–200 mg/day [47].

It is of interest that cells have the ability to operate their own “bypass” mechanisms in CoQ10 deficiency. Thus, the enzyme sulphide quinone oxidoreductase, which normally has a key role in sulphur metabolism, can transfer electrons directly to complexes III and IV of the mitochondrial respiratory chain, thereby bypassing complexes I and II and enabling some ATP production during CoQ10 deficiency [48]. However, in strict terminology, this process represents a compensatory mechanism rather than a bypass mechanism. Compensatory mechanisms are endogenous (often inadequate) responses of cells to counteract coenzyme Q10 deficiency, an example being an upregulation of glycolysis in an attempt to maintain the cellular supply of ATP. In contrast, bypass mechanisms are external pharmacological interventions providing supplementary CoQ10 or precursor compounds of the type described in this review to circumvent primary CoQ10 deficiency.

## 5. Conclusions

At present, most of the data relating to bypass mechanisms for CoQ10 deficiency have been derived from studies of yeast, animal models of CoQ10 deficiency, or cultured fibroblasts from patients with CoQ10 deficiency. Based on these studies, it has been demonstrated that supplementation with 4-HB can bypass COQ2 deficiency, supplementation with 2,4-DHB can bypass deficiencies in COQ7 and COQ9, supplementation with vanillic acid can bypass COQ6 and COQ9 deficiency, and supplementation with 3,4-DHB can bypass COQ6 deficiency, thereby improving CoQ10 levels, mitochondrial function and ATP generation. It is of note that preclinical studies in mice have provided evidence that 4-HB can access the BBB, thereby increasing brain CoQ levels. Thus, Corral-Sarasa et al. [6] demonstrated that administration of 4-HB increased CoQ9 levels in the brains of *COQ2* mice and that subsequent withdrawal of 4-HB resulted in a reduction in CoQ9 levels in the cerebrum and cerebellum. Similarly, Shi et al. [28] showed that 4-HMA and 4-HB are incorporated into CoQ9 and CoQ10 in the brains of *Hpdl*−/− mice, with a resultant increase in CoQ pools. In the present article, we have suggested the potential use of appropriate model systems to establish whether bypass compounds can access the BBB in man. Similarly, whilst 4-HB has been shown in animal models to cross the inner mitochondrial membrane [49], this has still to be demonstrated in man.

Preliminary clinical studies have reported significant symptomatic benefit following bypass treatments in individual patients with CoQ10 deficiency, suggesting that further clinical studies are now warranted. A limitation of this area of clinical research is that the rarity of the primary CoQ10 disorders precludes conventional randomised controlled trials to establish the efficacy and safety of such bypass treatments. However, since the report of the successful bypass treatment of a patient with COQ2 deficiency by Distelmaiert et al. [8], a further report from the same research group on the successful treatment of a second COQ2 patient using a similar bypass strategy has now been published [50]. In this study, 4-HB supplementation was initiated in a neonate with the severe neonatal onset phenotype of COQ2 deficiency (in which initial CoQ10 supplementation had appeared to be of no benefit); severe lactic acidosis was normalised, and normal renal function recovered. This study also demonstrated the absence of adverse effects of 4-HB supplementation (up to 166 mg/kg/day in divided doses) in a neonate, as well as in the 3-year-old previously described [8]. It is also of note that in the report by Munch et al. [50], a previous child from the same family who also had the same severe neonatal onset form of COQ2 deficiency subsequently died aged 2 ½ months; in this patient, supplementary CoQ10 was of no benefit, and bypass therapy with 4-HB was not available. Such studies provide a rationale for further clinical research to evaluate the efficacy of the preclinical bypass strategies identified in the present review in patients with relevant forms of primary CoQ10 deficiency. At present, no active or proposed clinical trials supplementing precursors such as 4-HB analogues for primary CoQ10 deficiency have been identified on any of the major trial registries (Clinical Trials.gov, ISRCTN, EU-CTR/CTS, and WHO ICTRP), although the FDA has given approval for the use of 4-HB in single patients with HPDL deficiency. In terms of future directions of this area of research, a key issue is the further investigation of the potential mechanisms by which bypass compounds such as 4-HB can access the human BBB; to further facilitate this issue, the use of a co-culture BBB model system using CoQ10-deficient neurons could be employed to assess the ability of bypass molecules to replenish cellular CoQ10 status. Further issues that should also be addressed include improving the identification of patients earlier via newborn screening to apply bypass therapies before permanent tissue damage occurs, further confirming the safety of bypass substances in humans, and further investigating how bypass strategies could also benefit secondary Q10 deficiencies, such as those reported in Parkinson’s or Alzheimer’s disease.

## Data Availability

No new data were created or analyzed in this study. Data sharing is not applicable to this article.
